# Age at Death of Creutzfeldt-Jakob Disease in Subsequent Family Generation Carrying the E200K Mutation of the Prion Protein Gene

**DOI:** 10.1371/journal.pone.0060376

**Published:** 2013-04-02

**Authors:** Maurizio Pocchiari, Anna Poleggi, Maria Puopolo, Marco D’Alessandro, Dorina Tiple, Anna Ladogana

**Affiliations:** Department of Cell Biology and Neurosciences, Istituto Superiore di Sanità, Rome, Italy; The Scripps Research Institute Scripps Florida, United States of America

## Abstract

**Background:**

The E200K mutation of the prion protein gene (*PRNP*) is the most frequent amino acid substitution in genetic Creutzfeldt-Jakob disease and is the only one responsible for the appearance of clustered cases in the world. In the Israel and Slovakian clusters, age of disease onset was reduced in successive generations but the absence of a clear molecular basis raised the possibility that this event was an observational bias. The aim of the present study was to investigate possible selection biases or confounding factors related to anticipation in E200K CJD patients belonging to a cluster in Southern Italy.

**Methods:**

Clinical and demographical data of 41 parent-offspring pairs from 19 pedigrees of the Italian cluster of E200K patients were collected. Age at death of parents was compared with age at death of E200K CJD offspring. Subgroup analyses were performed for controlling possible selection biases, confounding factors, or both.

**Results:**

The mean age at death/last follow-up of the parent generation was 71.4 years while that of CJD offspring was 59.3 years with an estimated anticipation of 12.1 years. When the same analysis was performed including only parents with CJD or carrying the E200K mutation (n = 26), the difference between offspring and parents increased to 14.8 years.

**Conclusions:**

These results show that early age at death occurs in offspring of families carrying the E200K *PRNP* mutation and that this event is not linked to observational biases. Although molecular or environmental bases for this occurrence remain unsettled, this information is important for improving the accuracy of information to give to mutated carriers.

## Introduction

The glutamine to lysine change at codon 200 (E200K) is the most common mutation of the prion protein (PrP) gene (*PRNP)* accounting for more than 70% of genetic prion diseases worldwide [1]–[3]. This mutation is responsible for all known clustered cases in the world [4]–[7], including that described in the Italo-Greek villages of the Southeast area of the Calabria region in Italy [6]. In this cluster, mutated carriers either develop Creutzfeldt-Jakob disease (CJD) with great age variability (from the third to the eighth decade) or do not develop disease at all resulting in a cumulative penetrance (67%) [8] similar to the Slovakian cluster (60%) [9] but lower than that reported in Libyan Jews in Israel (96%) [10]. In the past 20 years of CJD surveillance in the Calabrian cluster we observed a consistent early age of CJD onset in offspring compared to those of their relatives suggesting anticipation of disease onset similarly to those reported in E200K CJD Libyan Jews leaving in Israel [11] and in the Slovakian cluster [12]. The aim of this study was to formally analyze data on age at death of E200K CJD patients in the Calabrian cluster to account for possible selection biases or confounding factors.

## Methods

### Protocol Approvals, Registrations, and Patient Consents

Clinical data on patients, including family history and genetic analyses, were obtained following the criteria of the CJD surveillance program (approved by the Ethical Committee of the Istituto Superiore di Sanità) and data are stored in a database, which is registered at the Italian data protection Agency. Written informed consents for clinical data collection, blood sampling, and genetic analysis were obtained from patients (or their next-on-kin) and healthy subject involved in the study.

### Study Population and Data Collection

We studied 41 parent-offspring pairs from 19 apparently unrelated pedigrees of the E200K CJD Calabrian cluster. Parent generation consisted of 34 subjects because there were two (n = 5) or three (n = 1) CJD-affected siblings in the same family. Diagnosis and classification of genetic CJD patients were done by internationally established criteria [13].

Information on medical history and age at death (or age at the last follow-up for unaffected parents) were obtained as described in table 1. In 15 parent-offspring pairs we were unable to infer the CJD-transmitting parent. We then took the conservative approach of using the age at death (or last follow-up) of the young parent.

**Table 1:** Source of diagnostic information in offspring and parent subjects.

Diagnosis/Source	Offspring	Parent
Probable and Definite CJD/CJD Registry	35	7
Probable and Definite CJD/Medical records	4	2
Possible CJD/Family recollection or Municipal Registry Offices	2	3
E200K carriers with no CJD/CJD Registry	–	2
Obligate E200K carriers with no CJD/CJD Registry, Family recollection, or Municipal Registry Offices	–	7
Unknown parent carriers/Family recollection or Municipal Registry Offices	–	13
TOTAL^a^	41	34

a The discrepancy between the total number of offspring and parent is because two (n = 5) or three (n = 1) offspring had the same parent.

### *PRNP* Analysis

We performed the direct complete sequencing of the *PRNP* gene in 22 patients and 2 healthy carriers included in this study. Genomic DNA was extracted from whole blood using the QIAamp DNA KIT (QIAGEN), according to the manufacture’s recommendations. The *PRNP* open reading frame (ORF) was amplified by the PCR as previously described [8]. Referral clinicians provided genetic data on the other 9 CJD patients included in the study.

### Statistical Analysis

We assessed anticipation as differences between the age at death (or age at last follow-up) of parents and those of their offspring by one-tailed paired t-test to account for within pair data dependency. The paired differences for each pair were used to estimate anticipation. In the calculation of overall statistics, parents’ data were repeated for each offspring included in the study. We used age at death rather than age at onset because the latter is more difficult to ascertain in parental generation where data are mainly collected retrospectively. Moreover, the short disease duration of E200K patients (median 5 months) makes age at onset similar to age at death.

The influence of possible selection biases in data collection, confounding factors, or both was investigated by re-analyzing anticipation data by subgroups of pairs defined by different variables: gender, to control for sex-specific differences in mortality rates for genetic CJD in Italy [8]; polymorphism at codon 129 of the *PRNP* gene, to control for differences in the presentation of disease [1] and in disease duration [14]; birth cohort, to control for differences in the length of available follow-up or possible different exposure to causative or environmental agents; death cohort, to control for improvement in surveillance performance, diagnostic accuracy, or alertness to CJD; age at death, to investigate differences in anticipation between old cases - whose parents likely had enough time to develop CJD - and young cases - whose parents might develop the disease after their descendants; and parent’s age at death, to investigate the statistical phenomenon of regression to the mean [15], which states that anticipation is evident only in offspring of old parents. Subgroups for continuous covariates were obtained by splitting samples according to the observed median values (year 1937 for birth cohort; year 2000 for death cohort, age 61 years for age at death; and age 70 years for parents’ age at death or age at last evaluation).

Data are summarized by mean, standard deviation (SD) of the mean, and ranges. Observed p-values of paired t-test are reported. Comparisons were carried out at a significance level of 0.05 with no adjustment for multiple comparisons because of the explorative approach of the subgroups analyses. Data are graphed by box-plots.

## Results

Demographic and clinical characteristics of E200K CJD patients included in the study are reported in table 2. Prospective and retrospective CJD patients had similar sex distribution, age at death, and disease duration suggesting that data taken retrospectively were accurate. As expected because codon 200 mutation in the Calabrian cluster co-segregates with methionine at codon 129, more than 75% of patients were methionine homozygous.

**Table 2:** Demographic and clinical characteristics of E200K CJD patients included in the study.

CJD cases	Sex			*PRNP* polymorphism at codon 129		Age at death			Disease duration (months)		
	M	F	p^a^	MM	MV	Mean, SD	range	p^b^	Median	range	p^b^
**Prospective**	14	22	1	23	7	59.6, 11.1	39–87	0.58	4.5	2–24	0.97
**Retrospective**	4	5		–	–	61.8, 7.30	49–73		6 (n = 6)	2–16	

a Fisher’s exact test.

b t-test.

**Figure 1:**
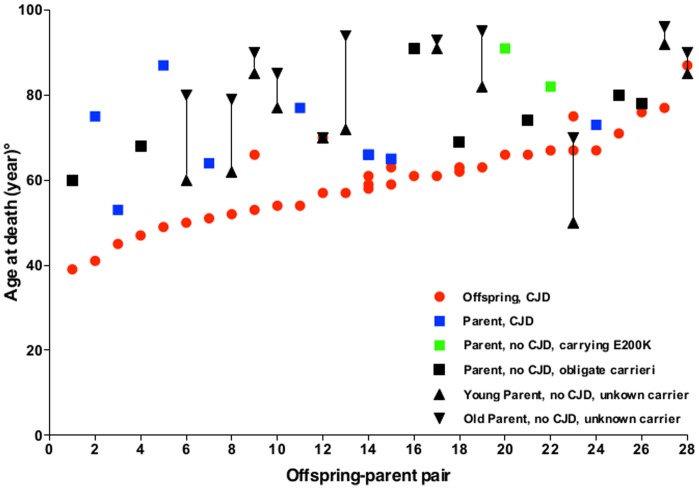
Parent-offspring pairs in the study population. Black circles, offspring with CJD; black squares, parents with CJD; black and white squares, unaffected parents carrying the E200K *PRNP* mutation; white squares, unaffected parents who are obligate carriers for the E200K *PRNP* mutation; white triangles, the oldest (up) and youngest (down) unaffected parents where it was unfeasible to discriminate the carrier. Vertical bars link the age between the oldest and youngest unaffected parents where it was unfeasible to discriminate the carrier.

**Figure 2:**
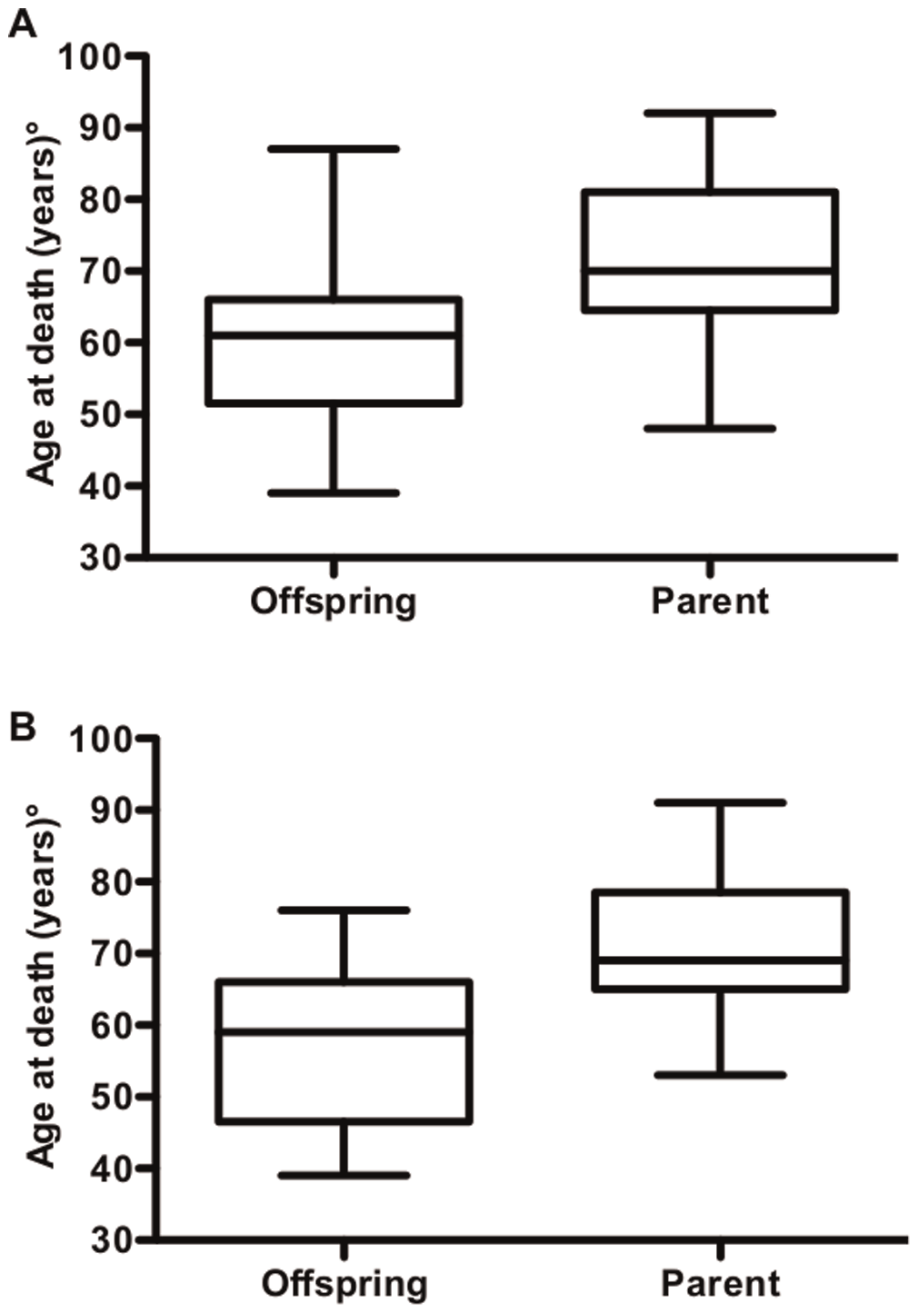
Age at death of offspring and parent generation. Box plots representing the age at death (or at the last clinical evaluation) for all offspring/parent pairs (a) or for pairs where parents had CJD, carried the E200K mutation, or were obligate carrier (b). The boxes extends from the 25th to the 75th percentile, bars extends to upper and lower adjacent values, and the lines in the middle of boxes represent the median value.

Figure 1 shows that in 36 of 41 pairs (88.0%) offspring died of CJD earlier than their parent. Overall, mean age at death of CJD offspring was 59.3 years (SD = 10.6; Range: 39–87) while that of the parent generation was 71.4 years (SD = 11.8; Range: 48–92) with an anticipation of 12.1 years (SD = 13.1; paired t-test, p<0.0001) (Figure 2a). This value, however, underestimates the lag of anticipation because when both parents did not show signs of CJD (n = 15) we selected that with the younger age at death or at last evaluation. When the same analysis was performed with the more relevant group, i.e., including only parents with CJD, carrying the E200K mutation, or obligate carriers for the E200K mutation (n = 26), the difference between offspring and parents increased to 14.8±9.93 (p<0.0001) (Figure 2b).

In this restricted group we also analyzed the role of possible selection biases, confounding in data collection, or both (Table 3). Anticipation was statistically significant in all subgroups, but it was more marked in the group of pairs where offspring was born after 1938, where parents died at ≥70 years, and where offspring died at <61 years. The finding that anticipation was statistically significant in the group of pairs where the age at death (or at last evaluation) was relative early (<70 years) excludes the possibility that the observed anticipation is related to the so-called phenomenon of regression to the mean [15].

**Table 3:** Analyses of possible selection biases or confounding factors by offspring covariates.

	Number of pairs	Anticipation (years)Mean, SD	p-value^a^
**Overall**	26	14.7, 9.90	<0.0001
***Gender***			
Male	11	14.1, 10.6	0.0006
Female	15	15.3, 9.27	<0.0001
***Codon 129 polymorphism*** b			
** **Met/Met	17	13.9, 10.4	<0.0001
***Birth cohort***			
<1939	10	9.70, 7.86	0.0018
≥1939	16	18.0, 9.89	<0.0001
***Death cohort***			
<2000	11	15.6, 12.6	0.0011
≥2000	15	15.4, 8.95	<0.0001
***Age at death***			
<61 years	14	17.7, 9.80	<0.0001
≥61 years	12	11.2, 9.20	0.0007
**Parent’s age**			
<70 years	14	11.4, 6.96	<0.0001
≥70 years	12	18.6, 11.6	<0.0001

a one tailed paired t-test.

b Four patients were Met/Val at codon 129.

**Figure 3:**
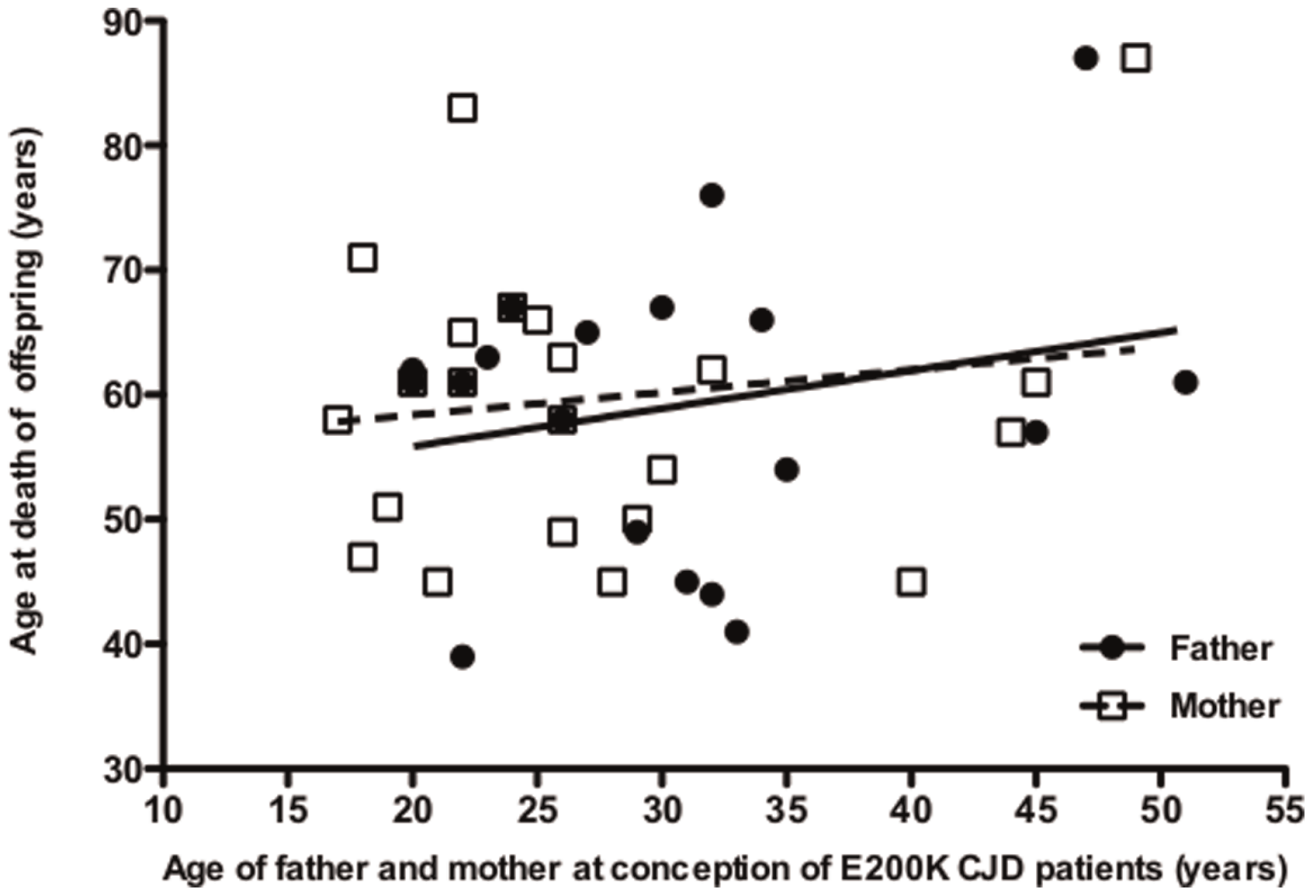
Father’s and mother’s age at conception versus age at death of offspring. The solid (father) and dashed (mother) lines denote the linear fit of data.

Finally, the influence of father’s age at conception, as an indirect indicator of *de novo* genetic mutations, and mother’s age at conception, as internal control, did not show any significant correlation with age at onset of disease (Figure 3).

## Discussion

In affected E200K CJD families belonging to the Calabrian cluster, offspring develop disease about 12 years earlier than their parents similarly to what was previously reported in other E200K CJD clusters in Israel [11] and Slovakia [12]. All together, these data reveal that anticipation of disease onset occurs in families affected with the E200K CJD. The term anticipation was set aside by Lionel Penrose, who claimed that anticipation was a statistical artifact arising as result of ascertainment biases [16], particularly in diseases, such as CJD, where the molecular basis of the phenomenon is still unexplained [17], [18]. Several ascertainment biases might contribute to the observation of anticipation, including the selection of parents with late onset of disease, the selection of offspring with early onset of disease, the selection of cases with simultaneous onset in parents and offspring, or any bias that would cause a truncation in reporting cases within a family [19]–[20]. An optimal study design should consider only prospective cases, but this is impractical in CJD and other rare diseases with onset in adult age [21]. Retrospective studies, on the other hand, suffer from possible ascertainment, confounding, or both biases, but these can be minimized and adjusted for by statistical analyses. In our study, ascertainment biases in selection of cases [16] are highly unlikely because we systematically included all E200K cases belonging to the Calabrian cluster and referred to the Italian CJD Registry without any selection on positive or negative family history for TSE, age, or status as offspring or parent. Selection biases or confounding factors in data collection were investigated in an explorative approach by subgroups analyses in the restricted group where parents developed CJD or carried the E200K mutation. These cases were stratified according to gender, polymorphism at codon 129 of *PRNP*, birth cohort, death cohort, age at death, and parent’s age at death. Although with some minor differences, anticipation of disease remained statistically significant and relatively stables (between 9.7 and 18.6 years) in all subgroups, suggesting that the most common confounding factors do not influence the observed results and that anticipation is likely a biological phenomenon to be considered in E200K carriers.

What remain unknown are the pathogenic mechanisms of anticipation, which are unlikely related to the coding region of the *PRNP* gene but rather to the upstream regulatory regions of *PRNP*, other host genes, or environmental factors. In the *PRNP* coding region, the polymorphic codon 129 of the non-mutated allele does not influence age at onset of disease in E200K CJD patients [1] and other rare polymorphisms [22] would unlikely influence age at onset and be responsible for the anticipation phenomenon. On the other hand, polymorphisms in the upstream regulatory regions of *PRNP* may influence susceptibility and age at onset in genetic CJD as observed in sporadic CJD [23] and might therefore be a confounding factor in our study. Although it is unlikely that in all offspring/parent pairs the deleterious polymorphism is systematically present in the offspring generation, it would be of interest to search for such polymorphisms in E200K CJD patients and healthy carriers belonging to the Calabrian and other CJD clusters worldwide. Other genes may be involved in the anticipation phenomenon, but the few genome-wide association studies (GWAs) in human prion diseases have only identified risk loci with modest effects in determining susceptibility to one form of prion disease, i.e. variant or sporadic CJD, but not in others. Overall, these studies have been so far disappointing, but further genetic association studies in homogenous population, such as that of the Italo-Greek minority of the Calabrian cluster with high inbreeding coefficient [24], would need to be performed for investigating whether there are genes, other than *PRNP*, that may account for differences in age at onset in E200K CJD patients. The lack of correlation between the father’s age at conception and age at onset suggests that *de novo* genetic mutations do not play an important role in modulating the development of E200K CJD as reported for autism or other disorders [25].

An increased exposure to possible risk factors in the offspring generation may be also responsible for early age at onset of disease. In the last decades, the improvement of medical procedures in the cluster area may have exposed offspring carriers to possible iatrogenic factors. However, we could not find any known iatrogenic procedures, i.e., treatment with human-derived pituitary hormones, *dura mater* grafts, neurosurgery, or corneal transplants [26] in the medical history of E200K CJD patients and other potential medical procedures, such as general surgery or blood transfusion, recently associated to sporadic CJD, were not confirmed in genetic CJD patients [27]. It is also unlikely that the anticipation observed in the offspring generation results from an early diagnosis as we used age at death rather than age at onset for our analysis and because of the short median disease duration (less than 1 year).

Finally, changes of environmental factors in soil, drinking water, or food that have occurred in the last decades might have contributed to an increased susceptibility, thus an early age at onset, in the offspring generation. The increased concentration of some metal microcrystal pollutants, particularly barium stronzium and silver, in the soil of the Calabrain cluster area with respect to adjacent neighborhood was proposed as a responsible factor for the high incidence of familial CJD cases [28]. However, soil samples were only collected at the end of 2004 and no data on the concentration of these metals in the decades before 2004 are available to support this highly speculative hypothesis. Moreover, the finding that the anticipation phenomenon was also observed in pairs where parents lived in the cluster area and offspring emigrated in Northern Italy (14.1 years, n = 9) and that an anticipation (12.8 years) similar to that observed in Israel [11] is also present in Libyan Jews living in Italy (data not shown), weaken this possibility. Increased oxidative insults in offspring of E200K carriers with respect to their parents might also be responsible for the observed anticipation phenomenon. The substitution of glutamic acid to lysine makes the mutant PrPc more susceptible to oxidation and, once oxidized, likely more inclined to change conformation into PrP^TSE^ [29]. A recent study done in a new transgenic (Tg) mouse line expressing the E200K mutation and spontaneously developing prion disease [30] has shown that the exposure of these mice to copper, a redox active metal, in drinking water induces disease earlier than untreated mice [31]. These data suggest that the E200K mutant PrPc is likely less competent to protect cells from copper induced oxidation, which may finally lead to an acceleration of the misfolding process, hence to an early age at onset of disease. Assuming that humans would behave like mice, it remains to determine which life events have increased oxidative insults in offspring that did not in their parents. Moreover, a recent study in the E200K CJD cluster of Slovakia reported an unbalance of manganese/copper concentration in brain tissues of genetic CJD cases in comparison to controls suggesting that metal disequilibrium might act as exogenous co-factor for the development of disease [32], but great caution is needed in the interpretation of these data.

The anticipation phenomenon is worth of further investigation because, if confirmed, will improve the accuracy of information to pass to mutated carriers and would possibly assist physicians in taking the difficult decision to start a preventive therapy, when would be available, at the right time.
